# Suppressing Effect of 2-Nitrobenzaldehyde on Singlet Oxygen Generation, Fatty Acid Photooxidation, and Dye-Sensitizer Degradation

**DOI:** 10.3390/antiox7120194

**Published:** 2018-12-18

**Authors:** Mahdi Hajimohammadi, Atena Vaziri Sereshk, Clemens Schwarzinger, Günther Knör

**Affiliations:** 1 Faculty of Chemistry, Kharazmi University, G. C, Mofateh, Tehran 14911-15719, Iran; 2 Department of Chemistry, Faculty of Science, Central Tehran Branch, Islamic Azad University, Shahrak Gharb, Tehran 14778-93855, Iran; mahdi.hajimohammadi@jku.at; 3 Institute of Chemical Technology of Organic Materials, Johannes Kepler University Linz, 4040 Linz, Austria; clemens.schwarzinger@jku.at; 4 Institute of Inorganic Chemistry, Johannes Kepler University Linz, 4040 Linz, Austria

**Keywords:** singlet oxygen scavenger, 2-nitrobenzaldehyde, inhibition of fatty acid photooxidation, UV photoprotection, photostabilization

## Abstract

2-Nitrobenzaldehyde was found to efficiently block singlet oxygen generation in a series of different test samples upon exposure to UV and visible light under aerobic conditions. The effect of quenching singlet oxygen formation was monitored in the presence of 1, 4-diazabicyclo [2.2.2] octane (DABCO) acting as a well-known singlet oxygen scavenger. A comparison of different nitrobenzaldehyde isomers with other highly effective synthetic antioxidants used in the food industry such as butylated hydroxytoluene (BHT), butylated hydroxyanisole (BHA), tert-butylhydroquinone (TBHQ) revealed that the protection of materials from singlet oxygen decreases in the order of 2-nitrobenzaldehyde > DABCO > TBHQ > 3-nitrobenzaldehyde > BHA > 4-nitrobenzaldehyde > BHT. Upon addition of 2-nitrobenzaldehyde, the oxidation of fatty acids and the degradation of photosensitizers was found to be considerably diminished, which indicates that the presence of 2-nitrobenzaldehyde has a significant protective influence by restricting the singlet oxygen generation and photodegradation of dyes. Moreover, the compound turned out to display its highly suppressing effects on typical singlet oxygen-dependent reactions, such as fatty acid photooxidation and dye photosensitizer degradation, in a rather broad spectral region covering wavelengths from 300 nm (UV-B) to 575 nm (close to the maximum of ambient solar radiation).

## Highlights:

- 2-Nitrobenzaldehyde acts as a photostabilizing agent against oxidative stress
- Suppression of singlet oxygen-dependent damage of substrates demonstrated
- Photoprotection of fatty acids exceeding that of well-established antioxidants
- Stabilization against self-sensitization and bleaching of irradiated organic dyes
- Possible beneficial effects on tissues by inhibition of lipid peroxidation expected

## 1. Introduction

In its electronic ground state (^3^Σ_g_^−^), molecular oxygen is characterized as a triplet diradical species carrying two unpaired electrons of parallel spin. Upon uptake of energy, these valence electrons may get paired together with opposite spin to generate a singlet oxygen molecule in the ^1^Δ_g_ excited state [1]. The electrophilic character of singlet oxygen may cause damage to lipids, amino acids, nucleic acids, and many other biological targets [2]. Under exposure to light, singlet oxygen can be easily generated in biological tissues and food systems, especially in the presence of endogenous photosensitizers such as riboflavin and chlorophylls [3,4,5]. Singlet oxygen attacks unsaturated fatty acids and produces lipid hydroperoxides as primary products of fatty acids [6]. Furthermore, exposure to UV radiation causes DNA damage and protein oxidation and induces the synthesis of matrix metalloproteinases [7]. The use of antioxidants to protect human skin from the harmful effects of UV radiation is therefore a topic that has attracted growing interest in recent years within the field of photoprotection research [8]. The nucleophilic amine 1,4-diazabicyclo [2.2.2] octane (DABCO) was recognized early as a very efficient quencher of singlet oxygen in organic media [9], and several synthetic antioxidants such as tert-butylhydroquinone (TBHQ), butylated hydroxyanisole (BHA), and butylated hydroxytoluene (BHT) have also been found to have a strong singlet oxygen quenching ability [10]. On the other hand, nitrobenzaldehyde and derivatives thereof are well known for their light-sensitivity and have also been demonstrated to display significant inhibitory effects on the activity of certain oxygen-dependent enzymes [11,12].

In this context, the present study was carried out to characterize the photoprotective potential of 2-nitrobenzaldehyde as an antioxidant in comparison with DABCO, a well-known benchmark singlet oxygen scavenger, and other highly effective antioxidants such as butylated hydroxyanisole (BHA), butylated hydroxytoluene (BHT), and tert-butyl hydroquinone (TBHQ). 

## 2. Materials and Methods

### 2.1. Material

Anthracene, oleic acid, linoleic acid, acetonitrile, methylene blue (MB), DABCO, BHT, BHA, TBHQ, 2-nitrobenzaldehyde, 3-nitrobenzaldehyde, and 4-nitrobenzaldehyde were purchased from Merck or Sigma-Aldrich (Vienna, Austria) and Alfa Aesar (Karlsruhe, Germany) without further purification. 5,10,15,20-Tetrakis(tolyl)porphyrin (H_2_TTP) and its ferric complex TTPFeCl were synthesized according to the literature [13,14].

### 2.2. Methods

#### 2.2.1. Sample Preparation for Anthracene Oxidation

In a typical experiment, 0.002 mmol of antioxidant was added to 7.5 mL of an acetonitrile solution containing anthracene (2 × 10^−4^ M) together with H_2_TTP (2.5 × 10^−5^ M), TTPFeCl (2.5 × 10^−5^ M), or MB (1 × 10^−4^ M) as a photosensitizer. Photolysis of the samples was carried out for 45 min at 298 K under atmospheric pressure and continuous bubbling with air to maintain a constant level of dissolved oxygen (ca. 8.1 mg/L assumed for 100% air saturation). A photochemical chamber reactor system (Rayonet RPR-100, Southern New England Ultraviolet Co Inc., Branford, CT, USA) equipped with a combination of 16 fluorescent lamps with emissions centered at 419 nm and 575 nm (8 × RPR-4190 and 8 × RPR-5750) was used as the irradiation source. Bleaching of the anthracene absorption bands was monitored at the 375 nm peak with a Cary 300 UV-Vis spectrophotometer (Varian Instruments, Walnut Creek, CA, USA).

#### 2.2.2. Sample Preparation for the Photooxidation of Fatty Acids

Samples were prepared by adding 1 mmol of antioxidant to 7.5 mL of an acetonitrile/acetone (2:1 *v*/*v*) solution containing oleic acid (6.7 × 10^−2^ M) or linoleic acid (6.7 × 10^−2^ M) and MB (2.6 × 10^−3^ M) as a photosensitizer. Photolysis of the samples was carried out for 45 min at 298 K under atmospheric pressure and continuous bubbling with air to maintain a constant level of dissolved oxygen (ca. 8.1 mg/L assumed for 100% air saturation). A photochemical chamber reactor system (Rayonet RPR-100) equipped with a combination of 16 fluorescent lamps with emissions centered at 419 nm and 575 nm (8 × RPR-4190 and 8 × RPR-5750) was used as the irradiation source. The consumption of the starting fatty acids was monitored by GC-MS using a Thermo Finnigan trace gas chromatograph coupled to a Fisons Instruments MD 800 mass spectrometer (Thermo Scientific, Waltham, MA, USA). 

#### 2.2.3. Sample Preparation for Degradation of Photosensitizers via Singlet Oxygen

Samples were prepared by adding 0.5 mmol of antioxidant to 7.5 mL of an acetonitrile solution of oleic acid (0.1 M) and MB (1 × 10^−4^ M). Photolysis of the samples was carried out for 45 min at 298 K under atmospheric pressure and continuous bubbling with air to maintain a constant level of dissolved oxygen (ca. 8.1 mg/L assumed for 100% air saturation). A photochemical chamber reactor system (Rayonet RPR-100) equipped with eight fluorescent lamps with emissions centered at 300 nm, 419 nm, or 575 nm (RPR-3000, RPR-4190, or RPR-5750) was used as the irradiation source. Gradual photosensitizer degradation was monitored by the bleaching of the 655 nm absorption band of MB with a Varian Cary 300 UV-Vis spectrophotometer.

## 3. Results and Discussion

### 3.1. Anthracene Photooxidation

**Figure 1:**
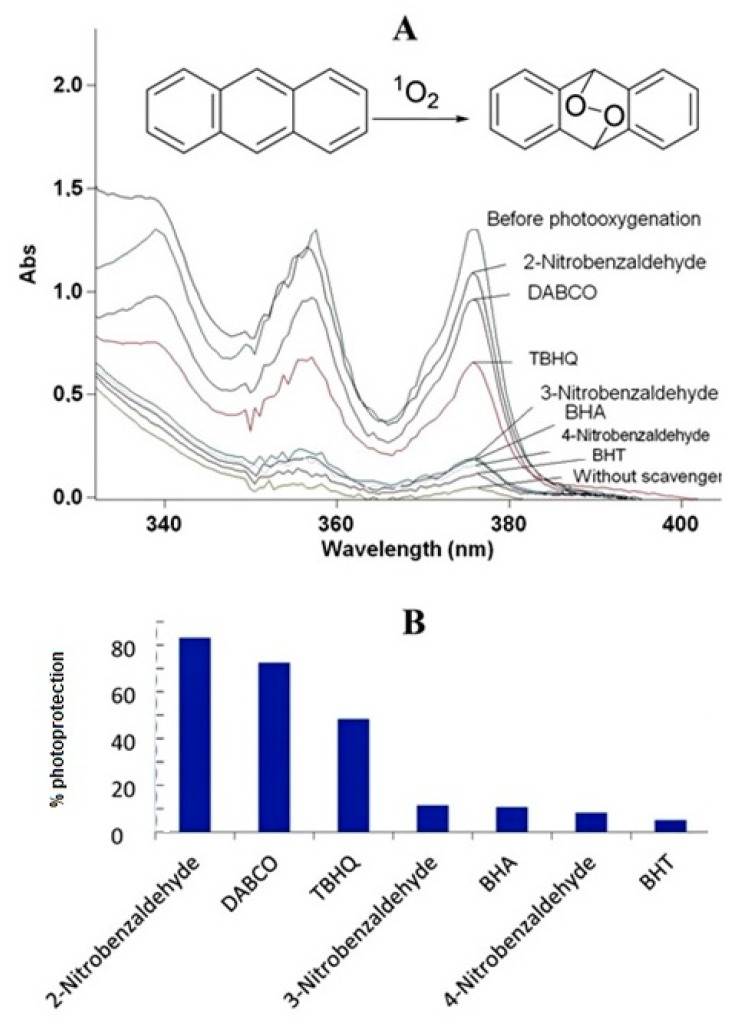
Variations of UV-Vis absorption spectra due to anthracene photooxidation after 45 min of exposure to methylene blue (MB) as a photosensitizer irradiated with a combination of 419 nm and 575 nm fluorescence lamp light in the absence and in the presence of different kinds of singlet oxygen scavengers (**A**). Comparison of the photoprotective capacity of different kinds of singlet oxygen scavengers after 45 min of irradiation of the anthracene containing samples under the conditions described above (**B**). DABCO: 1, 4-diazabicyclo [2.2.2] octane; TBHQ: tert-butylhydroquinone; BHA: butylated hydroxyanisole; BHT: butylated hydroxytoluene.

Electronic spectroscopy offers convenient methods for the direct or indirect detection of excited oxygen molecules. For spectrophotometric detection, a suitable chemical probe is usually applied to selectively trap singlet oxygen and enable its quantification based on absorption measurements. A very characteristic reaction suitable for following the photosensitized generation of singlet oxygen is the [4 + 2] cycloaddition with conjugated cyclic dienes or polycyclic aromatic hydrocarbons forming endoperoxide species with modified spectroscopic properties [15,16]. Anthracene is well known to reversibly trap singlet oxygen by such a cycloaddition reaction accompanied by characteristic spectral variations. In the present study, the generation of singlet oxygen induced by photoexcited methylene blue (MB) was evidenced by chemical trapping of ^1^O_2_ with anthracene. The UV-Vis spectral variations of anthracene as a function of irradiation time by use of MB as the photosensitizer are displayed in Figure 1A. A decrease of the absorption bands of anthracene (monitored at λ_max_ = 375 nm) was observed with increasing irradiation time. This spectral response can be interpreted as a consequence of the anthracene-9,10-endoperoxide formation interrupting the conjugated π-electron system of the polycyclic aromatic molecule (see Figure 1). It could be shown that the addition of DABCO, BHT, BHA, TBHQ, or 2-nitrobenzaldehyde as a novel antioxidant was able to inhibit the oxidation of anthracene in the order of 2-nitrobenzaldehyde > DABCO > TBHQ > 3-nitrobenzaldehyde > BHA > 4-nitrobenzaldehyde > BHT, as depicted in Figure 1A,B. Moreover, the bleaching reaction did not occur under dark control conditions, which is in agreement with the assumption that the anthracene oxidation requires the formation of singlet oxygen under visible light irradiation of MB. In summary, these results indicate that 2-nitrobenzaldehyde can act as a very effective suppressor of singlet oxygen generation or at least that it prevents substrates such as anthracene from being rapidly oxidized by the characteristic singlet oxygen mediated reactions. 

In Table 1, the protection from bleaching of the absorption bands of anthracene in the presence of different amounts of 2-nitrobenzaldehyde is presented. Remarkably, even at rather low concentrations of 2-nitrobenzaldehyde approaching the nanomolar range, the generation of singlet oxygen appears to be significantly inhibited (Table 1, entry 3). Moreover, upon variation of the types of photosensitizers applied (all of which having quite different spectral properties), the effect of 2-nitrobenzaldehyde on singlet oxygen generation under otherwise identical irradiation conditions in some cases was clearly of comparable magnitude, as observed in the presence of H_2_TTP instead of MB (Table 1, entry 4). The comparably low protective effect of 2-nitrobenzaldehyde in the presence of the metalloporphyrin photosensitizer tested (Table 1, entry 5) in contrast might indicate that besides the singlet oxygen-dependent bleaching pathways other routes of photochemical degradation of the organic substrate such as charge transfer-dependent photoredox processes [17] could also be operating when TPPFeCl-containing solutions are irradiated in the visible spectral region.

### 3.2. Reaction of Fatty Acids with Singlet Oxygen

Photosensitized generation of singlet oxygen under exposure to visible light has significance for many diverse research fields such as biomedical applications [16], selective organic transformations [18], and food chemistry [1,2,6]. Photooxidation of oleic acid as one of the exemplary targets of singlet oxygen was investigated as a typical standard sample to evaluate the potential antioxidant effect of 2-nitrobenzaldehyde in the context of lipid peroxidation (Table 2, entries 1 and 3). It is important to note that the oxidation of oleic acid stopped in the absence of MB or when the irradiation was interrupted. Therefore, the presence of a photosensitizer, light, and O_2_ are essential for the conversion oleic acid to the corresponding products. Oxidation of linoleic acid as a reactive fatty acid similarly was limited in the presence of 2-nitrobenzaldehyde (Table 2, entries 2 and 4). Furthermore, even low concentrations of 2-nitrobenzaldehyde have shown a respectable effect on the preservation of oleic acid and other unsaturated fatty acids from generating photooxidation products (Table 2, entries 5 and 6).

**Figure 2:**
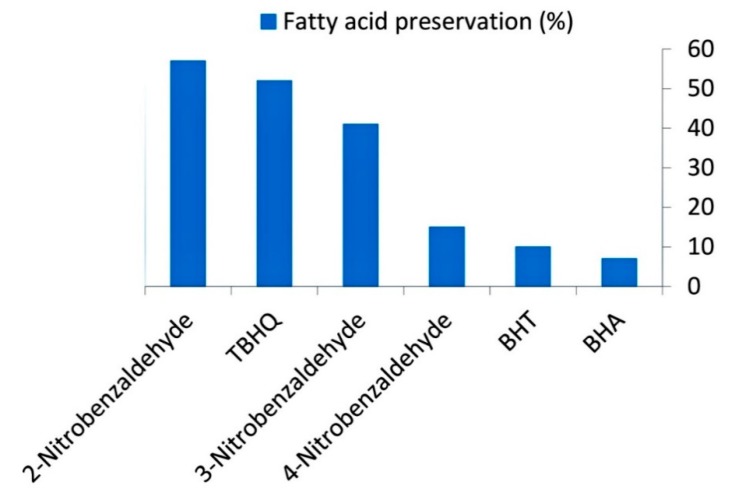
Diagram of fatty acid preservation (using oleic acid as a model substrate) in the presence of nitrobenzaldehyde isomers and well-known fatty acid antioxidants (BHA, BHT, and TBHQ).

Figure 2 shows the conversion of oleic acid in an oxygen saturated solution of acetonitrile/acetone under visible light in the presence of different nitrobenzaldehyde isomers and well-known fatty acid antioxidants (BHA, BHT, and TBHQ). According to the GC-MS data obtained, the rate of conversion of oleic acid is strongly diminished in the presence of 2-nitrobenzaldehyde, which shows that it can be used as an effective additive to protect fatty acids from oxidative decomposition in the presence of light and air.

### 3.3. Photodegradation of MB

While various organic dye molecules in their triplet excited state can be efficient photosensitizers for the generation of singlet oxygen, in many cases they are also potential targets for this reactive oxygen species once formed [19]. Consequently, there may be experimental conditions where the photosensitizers used to generate singlet oxygen will themselves react to quench it in subsequent secondary processes. This could then lead to photobleaching and gradual sensitizer degradation. In this context, the interaction of dyes with molecular oxygen under the influence of light and also their long-term stability upon light exposure has been a matter of great interest in photosensitization research [19,20,21,22,23]. Our tests using methylene blue (MB) as a representative photobleachable organic dye clearly indicated that increasing concentrations of 2-nitrobenzaldehyde were able to significantly improve the stability of the photosensitizer under continuous light exposure (entries 1–4 in Table 3). In addition, the degree of MB protection from UV-B light by 2-nitrobenzaldehyde was more effective than that from visible light (Table 3 entries 1, 5, and 6). These observations only partially can be ascribed to the intrinsic UV-B absorption properties of nitrobenzaldehydes leading to an inner-filter effect, since the para-substituted derivative shows only a minor protective effect (Table 3, entry 8). Moreover, the presence of a nitro functional group in the ortho-position of benzaldehyde in general performed more effectively for the photoprotection of MB than those in meta- and para-positions (Table 3 entries 1, 7, and 8).

**Figure 3:**
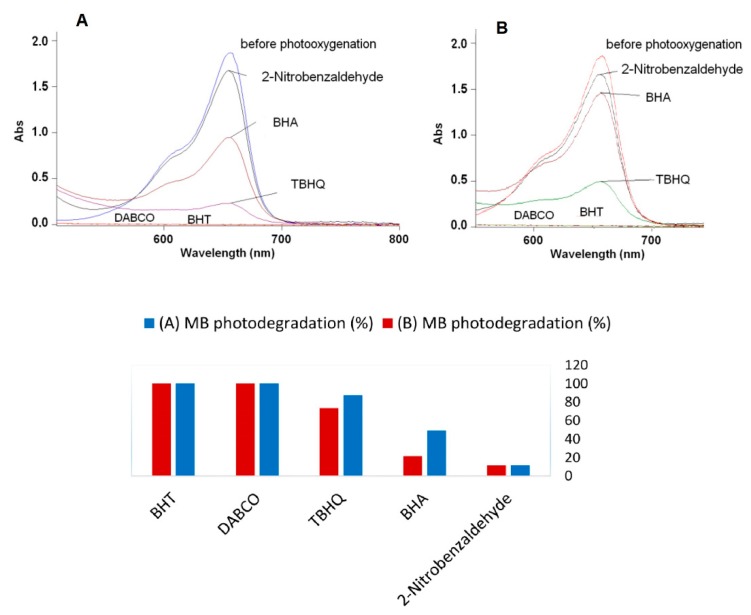
UV-Vis spectra and bar graph showing the degree of photodegradation of MB (λ_max_ = 655 nm) in the presence of different kinds of antioxidants after 24 h of irradiation with 16 fluorescent lamps (λ = 300 nm). Antioxidant concentrations: (**A**) 1 mmol DABCO, BHA, BHT, TBHQ, and 2-nitrobenzaldehyde. (**B**) 0.5 mmol DABCO, BHA, BHT, TBHQ, and 2-nitrobenzaldehyde.

It is important to point out that even at a reduced concentration of 2-nitrobenzaldehyde in the millimolar range, this compound maintained its photoprotective effect, and that the monitored MB degradation was significantly lower than with a double concentration of DABCO, BHA, BHT, and TBHQ (Figure 3). These results reveal that 2-nitrobenzaldehyde can act much more efficiently than various well-established singlet oxygen scavengers and photoprotective antioxidants currently applied for preventing dye degradation. Surprisingly, in the case of BHA and TBHQ, a higher concentration of these antioxidant additives obviously leads to a larger degree of degradation of the model substrate MB (Figure 3). This might indicate that light-generated decomposition products of BHA and TBHQ might induce destructive secondary dark reactions or radical-based processes leading to the observed MB bleaching effects under the reaction conditions selected here. 

**Scheme 1:**
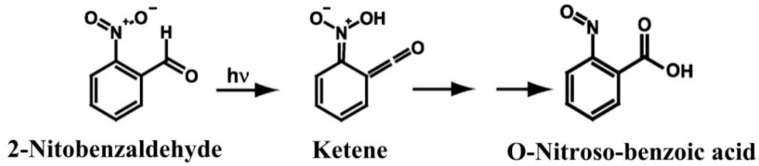
Proposed mechanism of the primary 2-nitrobenzaldehyde decomposition steps following UV-excitation [24], which are tentatively suggested here to be contributing to the observed strong photoprotective effects of this compound against UV-B light exposure of organic substrates such as unsaturated fatty acids or dye molecules.

An elucidation of the possible mechanistic aspects that could explain the extraordinary beneficial effects of 2-nitrobenzaldehyde on the photoprotection of unsaturated fatty acids and organic dye molecules was not within the scope of the present study. Nevertheless, it can be anticipated that the intramolecular primary processes occurring when 2-nitrobenzaldehyde is photoexcited with ultraviolet radiation might contribute a certain degree to the observations reported here (Scheme 1). According to the literature, 2-nitrobenzaldehyde is well known as a UV-photolabile compound, which is transformed into 2-nitroso-benzoic acid via a ketene intermediate and subsequent reaction steps with an overall quantum yield of Φ ~ 0.5, nearly regardless of the solvent [11,24]. It seems that the beneficial effects of 2-nitrobenzaldehyde as an additive for suppressing typical singlet oxygen-dependent photodegradation processes at least partially can be ascribed to its photolability and well-known intramolecular reactivity (Scheme 1).

## 4. Conclusions

Due to the increase of diseases such as cancer, skin disorders, Alzheimer’s disease, and other physiopathological effects related to the chemistry of reactive oxygen species (ROS) including singlet oxygen, the search for efficient new antioxidants is a very important research goal. Finding powerful photoprotective agents is also a challenging issue in the fields of food chemistry and material sciences. In this study, it could be clearly confirmed that 2-nitrobenzaldehyde performs very well as a photoprotective antioxidant and seems to play an effective role in restricting or limiting singlet oxygen generation, unsaturated fatty acid photooxidation and dye photodegradation. 

While an application of this antioxidant in the context of material photoprotection seems quite promising, it could not yet be shown here whether it will become possible to protect humans from singlet oxygen and to suppress the level of oxidative stress by administration of 2-nitrobenzaldehyde, especially due to the potential risk of undesirable side effects when larger amounts of the compound are metabolized in vivo [25]. Therefore, additional more detailed studies including the investigation of regular dose–effect relationships will be required to evaluate the potential for the future development of 2-nitrobenzaldehyde-based photoprotection strategies in the context of biomedical applications such as suppressing oxidative stress and lipid peroxidation in living cells and tissues.

## Figures and Tables

**Table 1:** Effect of 2-nitrobenzaldehyde on inhibition of singlet oxygen generation at different conditions.

Entry	Reaction Conditions	Inhibition of Singlet Oxygen Generation (%)
1 ^a^	2-nitrobenzaldehyde (0.002 mmol) + MB	83
2 ^a^	2-nitrobenzaldehyde (0.001 mmol) + MB	80
3 ^a^	2-nitrobenzaldehyde (0.0002 mmol) + MB	71
4 ^b^	2-nitrobenzaldehyde (0.002 mmol) + H_2_TTP	73
5 ^c^	2-nitrobenzaldehyde (0.002 mmol) + TTPFeCl	26

7.5 mL of acetonitrile solution containing anthracene (2 × 10^−4^ M), ^a^ MB (1 × 10^−4^ M), ^b^ H_2_TTP (2.5 × 10^−5^ M), ^c^ TTPFeCl (2.5 × 10^−5^ M), and air were irradiated by a combination of eight fluorescent lamps (λ = 419 nm) and eight fluorescent lamps (λ = 575 nm) for 45 min. Further details of these investigations can be found in the supplementary information (Figures S1–S4).

**Table 2:** Effect of 2-nitrobenzaldehyde on the preservation of unsaturated fatty acids from photooxidation ^a^.

Entry	Condition	Fatty Acid Preservation (%)
1	Oleic acid + 2-nitrobenzaldehyde	57
2	Linoleic acid + 2-nitrobenzaldehyde	54
3	Oleic acid	3
4	Linoleic acid	Trace
5 ^b^	Oleic acid + 2-nitrobenzaldehyde	51
6 ^c^	Linoleic acid + 2-nitrobenzaldehyde	23

^a^ 7.5 mL of acetonitrile/acetone (2/1 *v*:*v*), oleic acid and linoleic acid (6.7 × 10^−2^ M), MB (2.6 × 10^−3^ M), 2-nitrobenzaldehyde (1 × 10^−3^ mol), and air were irradiated by a combination of eight fluorescent lamps (λ = 419 nm) and eight fluorescent lamps (λ = 575 nm) for 48 h. ^b^ 1 × 10^−4^ mol 2-nitrobenzaldehyde. ^c^ 1 × 10^−5^ mol 2-nitrobenzaldehyde.

**Table 3:** MB degradation in the presence of singlet oxygen under different conditions.

Entry	Reaction Condition ^a^	MB Degradation (%)
1	2-nitrobenzaldehyde (0.5 mmol)	12
2	2-nitrobenzaldehyde (0.25 mmol)	30
3	2-nitrobenzaldehyde (0.05 mmol)	100
4	2-nitrobenzaldehyde (0.005 mmol)	100
5 ^b^	2-nitrobenzaldehyde (0.5 mmol)	23
6 ^c^	2-nitrobenzaldehyde (0.5 mmol)	22
7	3-nitrobenzaldehyde (0.5 mmol)	22
8	4-nitrobenzaldehyde (0.5 mmol)	98

7.5 mL of acetonitrile solution containing MB (1 × 10^−4^ M), oleic acid (0.1 M), and air were irradiated in a photoreactor (Rayonet RPR-100) with ^a^ eight fluorescent lamps (λ = 300 nm) ^b^ eight fluorescent lamps (λ = 419 nm) ^c^ eight fluorescent lamps (λ = 575 nm). Further details of these investigations can be found in the Supplementary Materials (Figures S5–S8).

## Supplementary Materials

The following are available online at http://www.mdpi.com/2076-3921/7/12/194/s1. Figure S1: Structures of the phenothiazine dye methylene blue (MB), as well as the *meso*-tetrakis (tolyl)porphyrin derivatives H_2_(TTP) and (TTP)FeCl applied as photosensitizers; Figure S2: UV-vis spectra revealing the degree of anthracene photooxygenation by singlet oxygen (analyzed at λ_max_ = 375 nm) in the presence of different concentrations of 2-nitrobenzaldehyde and MB as a photosensitizer after 45 min of visible-light irradiation using a combination of fluorescent lamps (maximum output at 419 nm and 575 nm); Figure S3: UV-vis spectra comparing the degree of anthracene photooxygenation by singlet oxygen (analyzed at λ_max_ = 375 nm) with H_2_(TTP) acting as a photosensitizer in the absence and presence of 2-nitrobenzaldehyde. Conditions: 45 min of visible-light irradiation using a combination of fluorescent lamps with lamps (maximum output at 419 nm and 575 nm); Figure S4: UV-Vis spectra comparing the degree of anthracene photooxygenation by singlet oxygen (analyzed at λ_max_ = 375 nm) with (TTP)FeCl as a photosensitizer in the absence and presence of 2-nitrobenzaldehyde. Conditions: 45 min of visible-light irradiation using a combination of fluorescent lamps with lamps (maximum output at 419 nm and 575 nm); Figure S5: UV-Vis spectra showing the photodegradation of MB (λ_max_ = 655 nm) in the presence of different nitrobenzaldehyde derivatives (1 mmol) after 24 h of UV-light irradiation with 300 nm fluorescent lamps; Figure S6: UV-Vis spectra showing the photodegradation of MB (λ_max_ = 655 nm) in the presence of different concentrations of 2-nitrobenzaldehyde (1.0 mmol, 0.5 mmol) after 24 h of UV-light irradiation with 300 nm fluorescent lamps; Figure S7: UV-Vis spectra showing the photodegradation of MB (λ_max_ = 655 nm) in the presence and in the absence of 2-nitrobenzaldehyde after 24 h of irradiation with 419 nm fluorescent lamps; Figure S8: UV-Vis spectra showing the photodegradation of MB (λ_max_ = 655 nm) in the presence and in the absence of 2-nitrobenzaldehyde after 24 h of irradiation with 575 nm fluorescent lamps; Figure S9: Photochemical reactor applied for the present study (https://rayonet.org/reactors.php?part=RPR-100).
