# Supplementary material for: Suppressing Effect of 2-Nitrobenzaldehyde on Singlet Oxygen Generation, Fatty Acid Photooxidation, and Dye-Sensitizer Degradation
Source: Antioxidants (Basel). 2018 Dec 18;7(12):194. doi: 10.3390/antiox7120194 (PMC6315359; doi:10.3390/antiox7120194)
Supplement: Supplementary file 1 [file antioxidants-07-00194-s001.pdf]

antioxidants

MDPI

Suppressing Effect of 2-Nitrobenzaldehyde on Singlet Oxygen Generation, Fatty Acid Photooxidation, and Dye-Sensitizer Degradation

Mahdi Hajimohammadi [1,*], Atena Vaziri Sereshk [2], Clemens Schwarzinger [3] and Günther Knör [4,*]

[1] Faculty of Chemistry, Kharazmi University, G. C, Mofateh, Tehran 14911-15719, Iran
[2] Department of Chemistry, Faculty of Science, Central Tehran Branch, Islamic Azad University, Shahrak Gharb, Tehran 14778-93855, Iran; mahdi.hajimohammadi@jku.at
[3] Institute of Chemical Technology of Organic Materials, Johannes Kepler University Linz, 4040 Linz, Austria; clemens.schwarzinger@jku.at
[4] Institute of Inorganic Chemistry, Johannes Kepler University Linz, 4040 Linz, Austria
* Correspondence: hajimohammadi@khu.ac.ir (M.H.); guenther.knoer@jku.at (G.K.)
Tel.: +98-21-888329220-3 (M.H.); +43-732-2468-5100 (G.K.); Fax: +98-21-88830857 (M.H.)

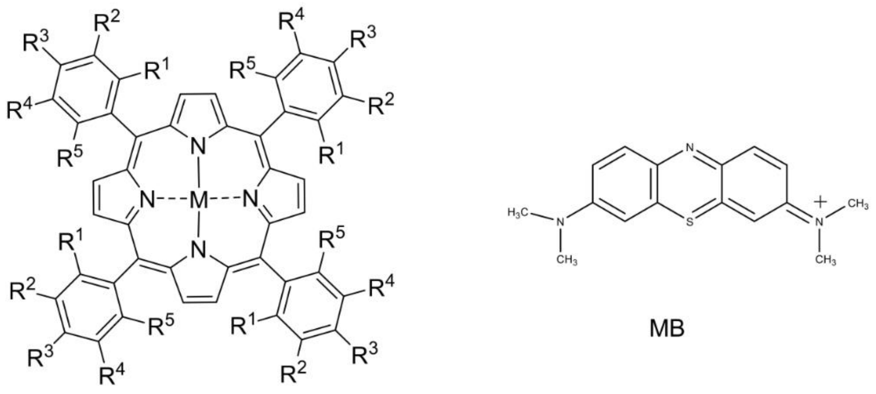

H_2TTP: R^1=R^2=R^4=R^5=H, R^3=Me,"M=H,H "
ClFeTTP: R^1=R^2=R^4=R^5=H, R^3=Me,"M=FeCl"

Figure S1. Structures of the phenothiazine dye methylene blue (MB), as well as the *meso*-tetrakis(tolyl)porphyrin derivatives H_2(TTP) and (TTP)FeCl applied as photosensitizers.

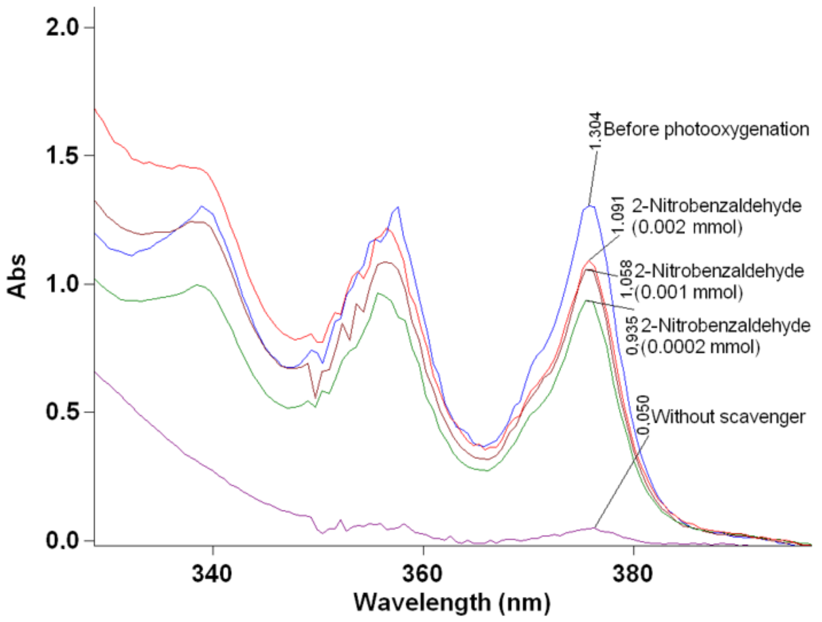

Figure S2. UV-vis spectra revealing the degree of anthracene photooxygenation by singlet oxygen (analyzed at λ_{max} = 375 nm) in the presence of different concentrations of 2-nitrobenzaldehyde and MB as a photosensitizer after 45 min of visible-light irradiation using a combination of fluorescent lamps (maximum output at 419 nm and 575 nm).

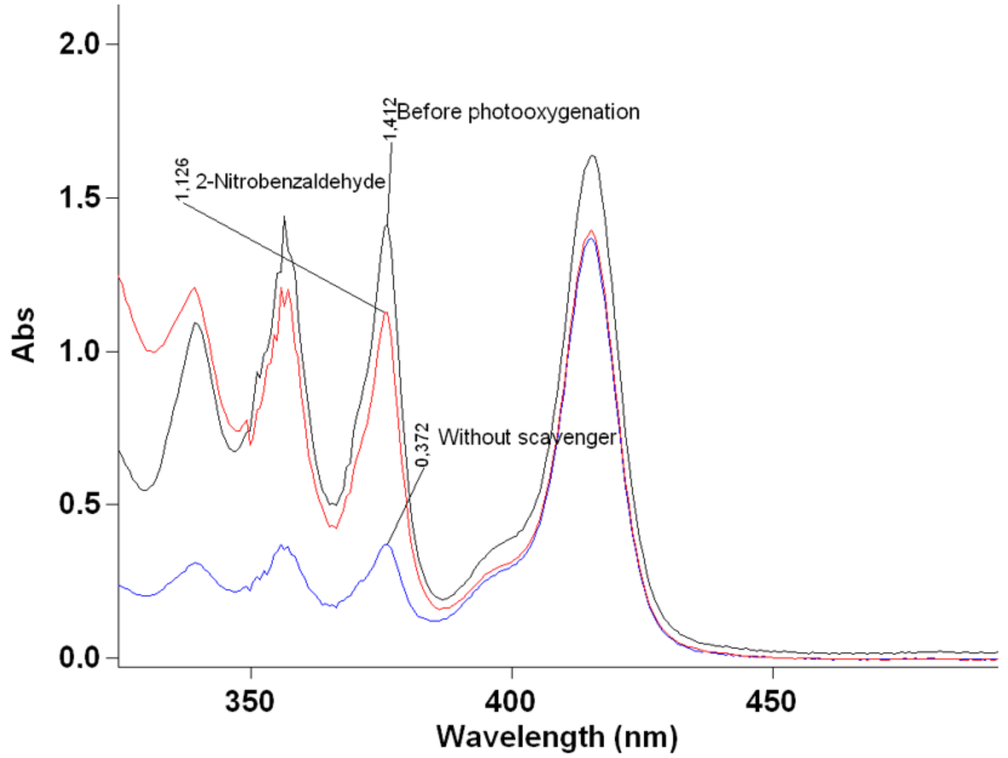

Figure S3. UV-vis spectra comparing the degree of anthracene photooxygenation by singlet oxygen (analyzed at λ_{max} = 375 nm) with H_2(TTP) acting as a photosensitizer in the absence and presence of 2-nitrobenzaldehyde. Conditions: 45 min of visible-light irradiation using a combination of fluorescent lamps (maximum output at 419 nm and 575 nm).

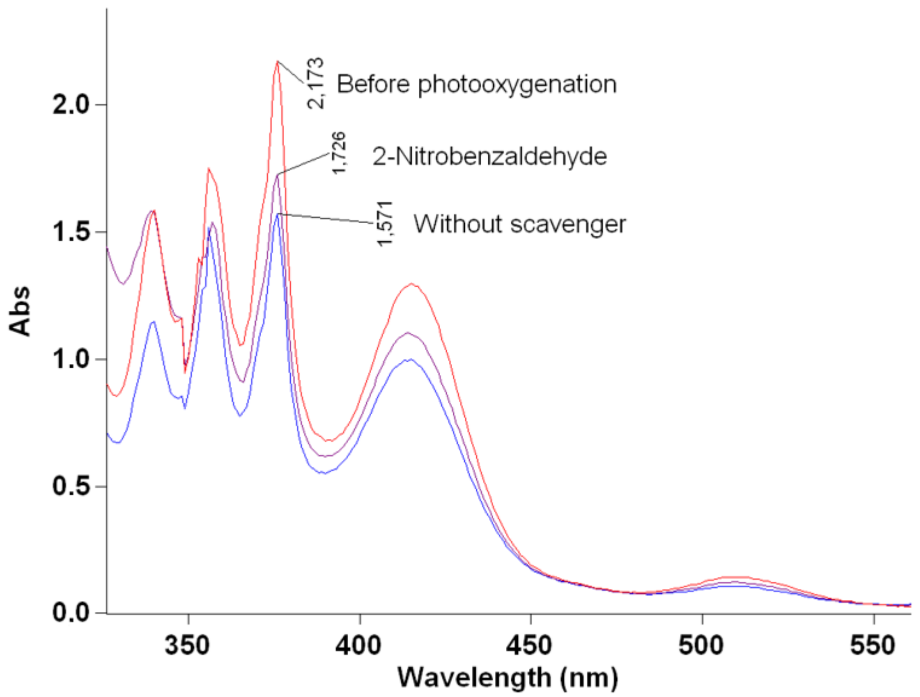

Figure S4. UV-Vis spectra comparing the degree of anthracene photooxygenation by singlet oxygen (analyzed at λ_{max} = 375 nm) with (TTP)FeCl as a photosensitizer in the absence and presence of 2-nitrobenzaldehyde. Conditions: 45 min of visible-light irradiation using a combination of fluorescent lamps (maximum output at 419 nm and 575 nm).

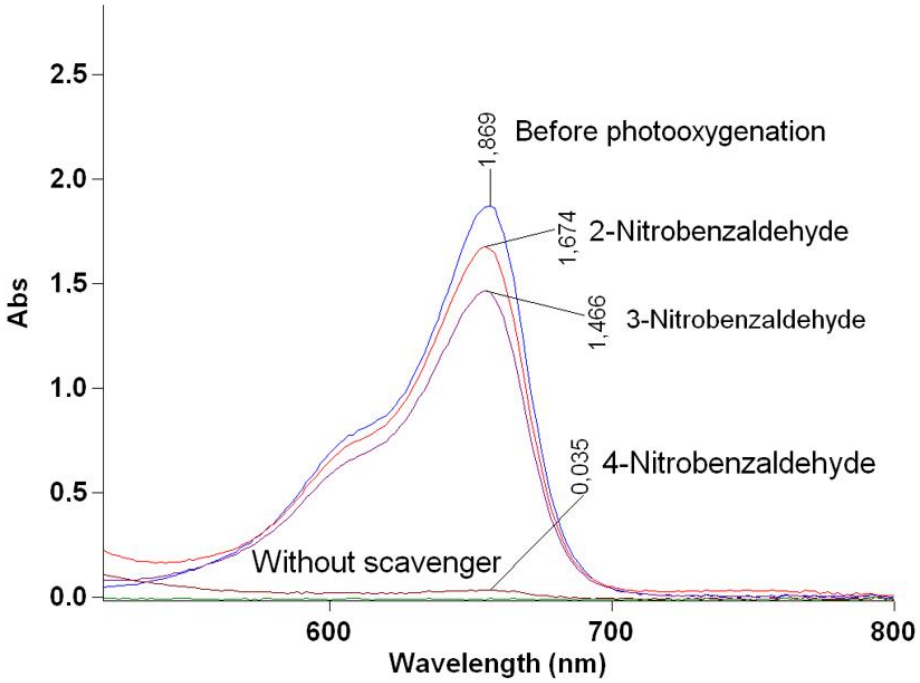

Figure S5. UV-Vis spectra showing the photodegradation of MB (λ_{max} = 655 nm) in the presence of different nitrobenzaldehyde derivatives (1 mmol) after 24 h of UV-light irradiation with 300 nm fluorescent lamps.

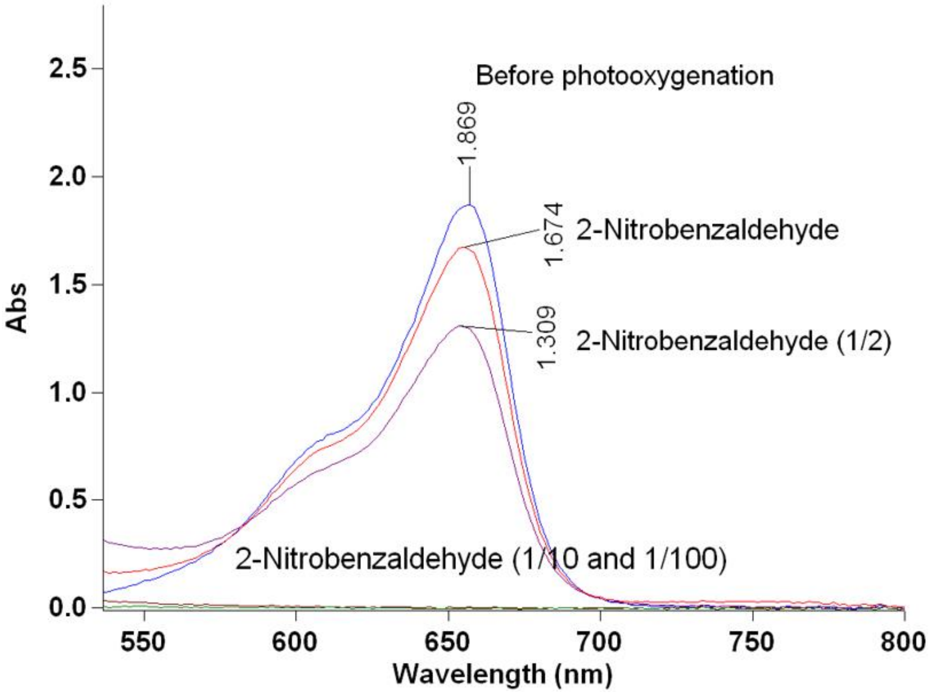

Figure S6. UV-Vis spectra showing the photodegradation of MB (λ_{max} = 655 nm) in the presence of different concentrations of 2-nitrobenzaldehyde (1.0 mmol, 0.5 mmol) after 24 h of UV-light irradiation with 300 nm fluorescent lamps.

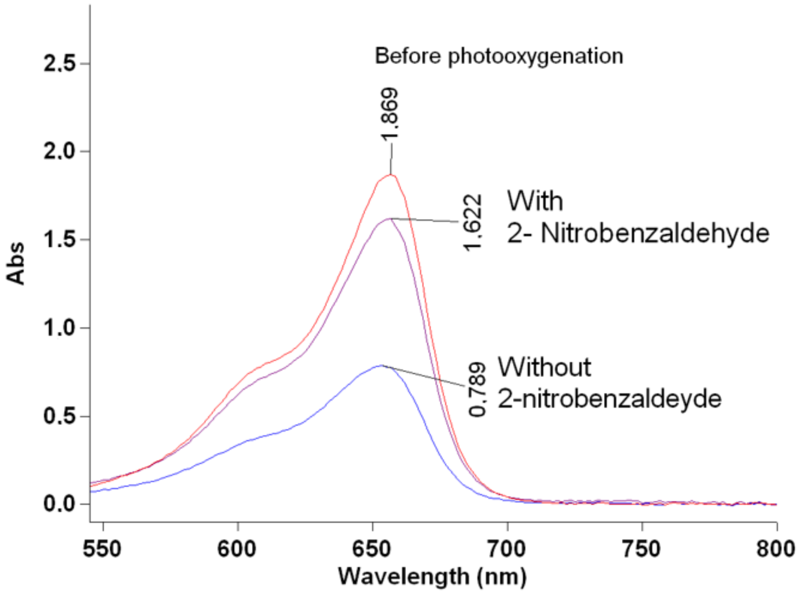

Figure S7. UV-Vis spectra showing the photodegradation of MB (λ_{max} = 655 nm) in the presence and in the absence of 2-nitrobenzaldehyde after 24 h of irradiation with 419 nm fluorescent lamps.

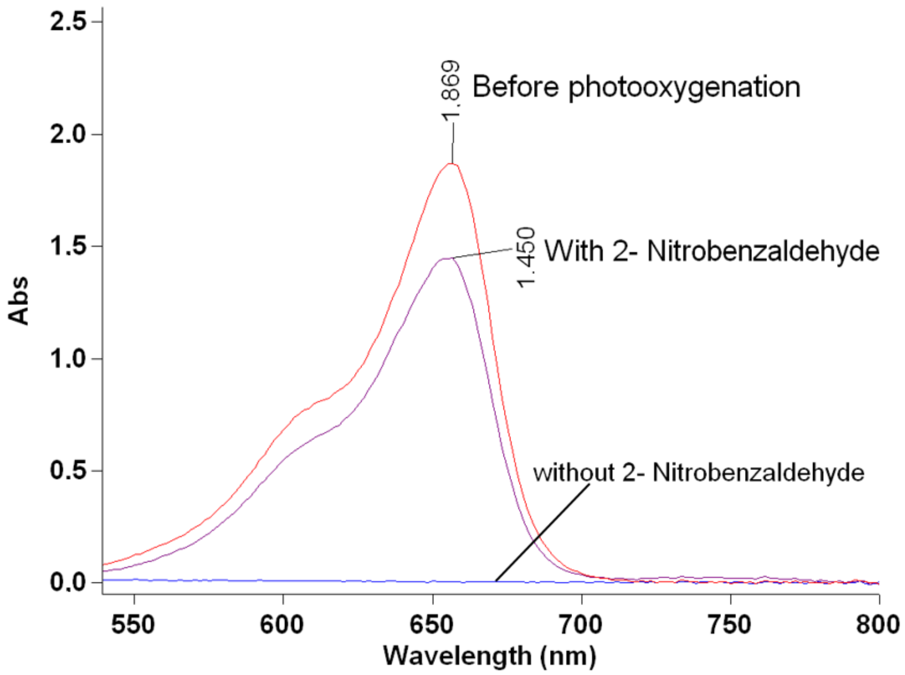

Figure S8. UV-Vis spectra showing the photodegradation of MB (λmax = 655 nm) in the presence and in the absence of 2-nitrobenzaldehyde after 24 h of irradiation with 575 nm fluorescent lamps.

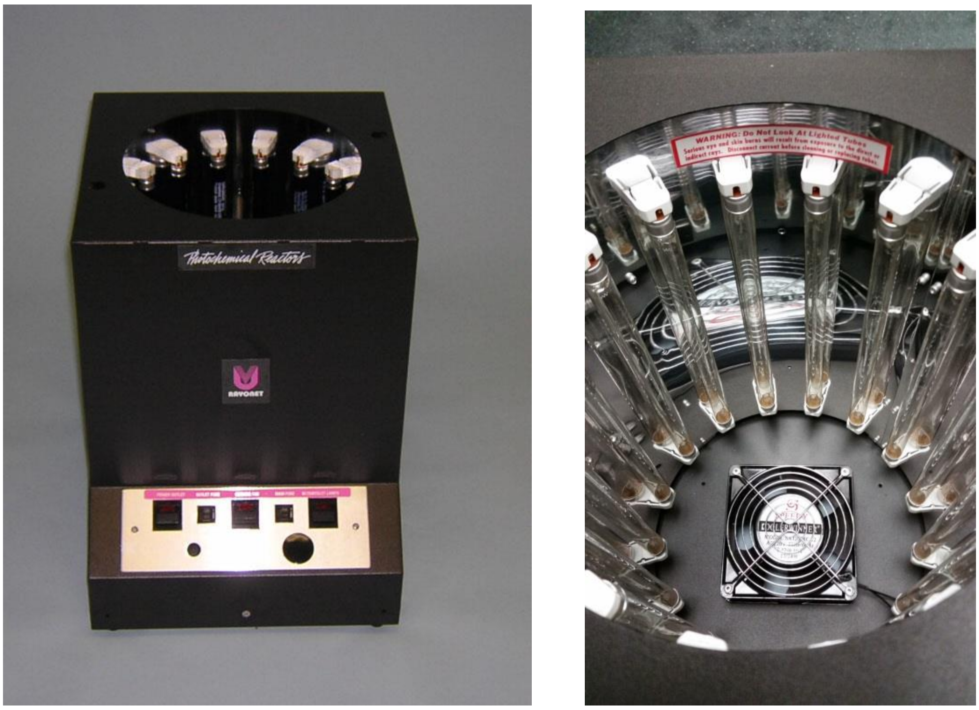

Figure S9. Photochemical reactor applied for the present study (https://rayonet.org/reactors.php?part=RPR-100).
